# Supplementary figures and images for: Case-control study of autonomic symptoms in the setting of Long COVID with tilt table testing
Source: PLoS One. 2025 Oct 24;20(10):e0335218. doi: 10.1371/journal.pone.0335218 (PMC12551903; doi:10.1371/journal.pone.0335218)

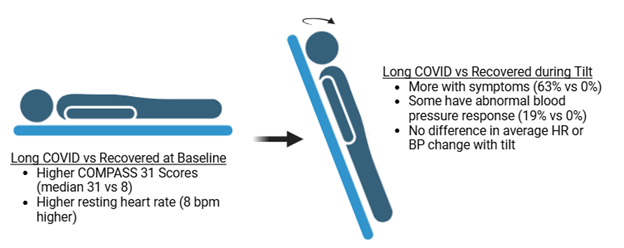

Supplement: S3 Fig — Among those with Long COVID compared to recovered comparator participants, we found higher COMPASS 31 scores, higher resting heart rate, more symptoms during head up tilt table testing, and that only some individuals meet diagnostic criteria for an abnormal hemodynamic response during tilt. (TIF) [file pone.0335218.s003.tif]
