# Supplementary material for: Case-control study of autonomic symptoms in the setting of Long COVID with tilt table testing
Source: PLoS One. 2025 Oct 24;20(10):e0335218. doi: 10.1371/journal.pone.0335218 (PMC12551903; doi:10.1371/journal.pone.0335218)
Supplement: Attachment — Submitted filename: Response to Reviewers.docx [file pone.0335218.s005.docx]

Response to Reviewers

==============================
**ACADEMIC EDITOR: Justify the study background**
==============================

**Authors’ Response:** We have added additional information to the introduction to justify the study background.

Journal Requirements:

When submitting your revision, we need you to address these additional requirements.

1. Please ensure that your manuscript meets PLOS ONE's style requirements, including those for file naming. The PLOS ONE style templates can be found at

[https://journals.plos.org/plosone/s/file?id=wjVg/PLOSOne_formatting_sample_main_body.pdf](https://urldefense.com/v3/__https:/journals.plos.org/plosone/s/file?id=wjVg*PLOSOne_formatting_sample_main_body.pdf__;Lw!!LQC6Cpwp!sqGHAxTi2C6t2-Xb-rykykSRTK2aFxyM8Bt5VMUuzCyaFHieJrJkZfuF76nCfujKuZdR1l-mcKVE1XGB_V3np0DE1w$) and

[https://journals.plos.org/plosone/s/file?id=ba62/PLOSOne_formatting_sample_title_authors_affiliations.pdf](https://urldefense.com/v3/__https:/track.editorialmanager.com/CL0/https:*2F*2Fjournals.plos.org*2Fplosone*2Fs*2Ffile*3Fid=ba62*2FPLOSOne_formatting_sample_title_authors_affiliations.pdf/1/010f01990e0e91cd-74e4c46e-b743-4c4d-8143-59488c57b46a-000000/Pi4kXUV0c_siUxaTnBD_TiAhXvVxD2eOszZENt0X970=227__;JSUlJSUlJQ!!LQC6Cpwp!sqGHAxTi2C6t2-Xb-rykykSRTK2aFxyM8Bt5VMUuzCyaFHieJrJkZfuF76nCfujKuZdR1l-mcKVE1XGB_V1rjkHRYw$).

**Authors’ Response:** We corrected the formatting and file naming. We did not find instructions for including a graphical abstract. Nonetheless, we have included a graphical abstract which can be removed at the editors’ discretion in case it can be included.

2. Thank you for stating in your Funding Statement:

“This study was primarily funded by a philanthropic gift from Charles W. Swanson to CL. MSD was funded by NIH/NHLBI K12 HL143961 and K23HL172699 (MSD). PYH was supported by NIH/NAID 2K24AI112393. The funders played no role in the study design, data collection and analysis, decision to publish, or preparation of the manuscript. Its contents are solely the responsibility of the authors and do not necessarily represent the official views of the NIH.”

Please provide an amended statement that declares *all* the funding or sources of support (whether external or internal to your organization) received during this study, as detailed online in our guide for authors at [http://journals.plos.org/plosone/s/submit-now](https://urldefense.com/v3/__http:/journals.plos.org/plosone/s/submit-now__;!!LQC6Cpwp!sqGHAxTi2C6t2-Xb-rykykSRTK2aFxyM8Bt5VMUuzCyaFHieJrJkZfuF76nCfujKuZdR1l-mcKVE1XGB_V14uoggRQ$).  Please also include the statement “There was no additional external funding received for this study.” in your updated Funding Statement.

Please include your amended Funding Statement within your cover letter. We will change the online submission form on your behalf.

**Authors’ Response:** We have included an amended statement which clarifies that additional funding was from internal division funds, and we now include the statement, “There was no additional external funding received for this study.”

3. Thank you for stating the following in the Competing Interests section:

“M.S.D: consulting fees from Merck. P.Y.H.: modest honoraria from Gilead and Merck and research grant from Novartis unrelated to the submitted work. M.J.P.: consulting fees from Gilead Sciences, AstraZeneca, BioVie, and Apellis Pharmaceuticals, and research support from Aerium Therapeutics, outside the submitted work. S.G.D.: consulting for Enanta Pharmaceuticals and Pfizer and research support from Aerium Therapeutics outside the submitted work. T.J.H.: consulting fees for Roche and Regeneron outside the submitted work. All other authors report no relevant disclosures or conflicts.”

We note that one or more of the authors are employed by a commercial company: Merck, Novartis, Gilead Sciences, AstraZeneca, BioVie, and Apellis Pharmaceuticals, Enanta Pharmaceuticals and Pfizer.

**Authors’ Response:** We would like to clarify that **none** of the authors are employed by a commercial company, although several of the authors have had limited consulting engagements with commercial entities outside of the submitted work, which we have been careful to disclose. We have included a slightly revised version in the cover letter to make it clear that none of the consulting engagements are related to the work reported in this manuscript.

a.        Please provide an amended Funding Statement declaring this commercial affiliation, as well as a statement regarding the Role of Funders in your study. If the funding organization did not play a role in the study design, data collection and analysis, decision to publish, or preparation of the manuscript and only provided financial support in the form of authors' salaries and/or research materials, please review your statements relating to the author contributions, and ensure you have specifically and accurately indicated the role(s) that these authors had in your study. You can update author roles in the Author Contributions section of the online submission form.

Please also include the following statement within your amended Funding Statement.

“The funder provided support in the form of salaries for authors [insert relevant initials], but did not have any additional role in the study design, data collection and analysis, decision to publish, or preparation of the manuscript. The specific roles of these authors are articulated in the ‘author contributions’ section.”

If your commercial affiliation did play a role in your study, please state and explain this role within your updated Funding Statement.

**Authors’ Response:** We would like to clarify that none of these commercial entities were involved at all in any way in this study, and these commercial entities did not employee nor pay the salaries of the investigators involved in this study. We have included a sentence about this in the revised cover letter.

b. Please also provide an updated Competing Interests Statement declaring this commercial affiliation along with any other relevant declarations relating to employment, consultancy, patents, products in development, or marketed products, etc.

Within your Competing Interests Statement, please confirm that this commercial affiliation does not alter your adherence to all PLOS ONE policies on sharing data and materials by including the following statement: "This does not alter our adherence to  PLOS ONE policies on sharing data and materials.” (as detailed online in our guide for authors [http://journals.plos.org/plosone/s/competing-interests](https://urldefense.com/v3/__http:/journals.plos.org/plosone/s/competing-interests__;!!LQC6Cpwp!sqGHAxTi2C6t2-Xb-rykykSRTK2aFxyM8Bt5VMUuzCyaFHieJrJkZfuF76nCfujKuZdR1l-mcKVE1XGB_V3IDhVxVw$)) . If this adherence statement is not accurate and  there are restrictions on sharing of data and/or materials, please state these. Please note that we cannot proceed with consideration of your article until this information has been declared.

Please include both an updated Funding Statement and Competing Interests Statement in your cover letter. We will change the online submission form on your behalf.

**Authors’ Response:** As described above, we have included updated funding statements and competing interests statements in the revised cover letter.

4. Thank you for stating the following in the Acknowledgments Section of your manuscript:

“This study was primarily funded by a philanthropic gift from Charles W. Swanson to CL. MSD was funded by NIH/NHLBI K12 HL143961 and K23HL172699 (MSD). PYH was supported by NIH/NAID 2K24AI112393. The funders played no role in the study design, data collection and analysis, decision to publish, or preparation of the manuscript. Its contents are solely the responsibility of the authors and do not necessarily represent the official views of the NIH. **“**

We note that you have provided additional information within the Acknowledgements Section that is not currently declared in your Funding Statement. Please note that funding information should not appear in the Acknowledgments section or other areas of your manuscript. We will only publish funding information present in the Funding Statement section of the online submission form.

Please remove any funding-related text from the manuscript and let us know how you would like to update your Funding Statement. Currently, your Funding Statement reads as follows:

“This study was primarily funded by a philanthropic gift from Charles W. Swanson to CL. MSD was funded by NIH/NHLBI K12 HL143961 and K23HL172699 (MSD). PYH was supported by NIH/NAID 2K24AI112393. The funders played no role in the study design, data collection and analysis, decision to publish, or preparation of the manuscript. Its contents are solely the responsibility of the authors and do not necessarily represent the official views of the NIH.”

Please include your amended statements within your cover letter; we will change the online submission form on your behalf.

**Authors’ Response:** We have removed these from the manuscript and noted them in the revised cover letter.

5. Please include captions for your Supporting Information files at the end of your manuscript, and update any in-text citations to match accordingly. Please see our Supporting Information guidelines for more information: [http://journals.plos.org/plosone/s/supporting-information](https://urldefense.com/v3/__http:/track.editorialmanager.com/CL0/http:*2F*2Fjournals.plos.org*2Fplosone*2Fs*2Fsupporting-information/1/010f01990e0e91cd-74e4c46e-b743-4c4d-8143-59488c57b46a-000000/GV3HW8nL1stxAAg0jOP-nMtBb3LEYp4hllY0jKCVI6Y=227__;JSUlJSU!!LQC6Cpwp!sqGHAxTi2C6t2-Xb-rykykSRTK2aFxyM8Bt5VMUuzCyaFHieJrJkZfuF76nCfujKuZdR1l-mcKVE1XGB_V1xlYSPLw$).

**Authors’ Response:** We have added captions to the supporting information and corrected the in-text citations and file names for the supporting information.

6. If the reviewer comments include a recommendation to cite specific previously published works, please review and evaluate these publications to determine whether they are relevant and should be cited. There is no requirement to cite these works unless the editor has indicated otherwise.

**Authors’ Response:** Not applicable, reviewers did not suggest citing specific work.

7. Please review your reference list to ensure that it is complete and correct. If you have cited papers that have been retracted, please include the rationale for doing so in the manuscript text, or remove these references and replace them with relevant current references. Any changes to the reference list should be mentioned in the rebuttal letter that accompanies your revised manuscript. If you need to cite a retracted article, indicate the article’s retracted status in the References list and also include a citation and full reference for the retraction notice.

**Authors’ Response:** We have reviewed the reference list; we have added several references in response to the Editor and Reviewer comments to expand the introduction/background. None of the references we have cited have been retracted.

Additional Editor Comments (if provided):

It is definitely an interesting article which will add value to the current research on COVID-19 studies. However, Please justify the background of the study and how tilt table test adds value.

**Authors’ Response:** Thank you for this comment. We have added additional background to the introduction.

[Note: HTML markup is below. Please do not edit.]

Reviewers' comments:

Reviewer's Responses to Questions

**Comments to the Author**

1. Is the manuscript technically sound, and do the data support the conclusions?

The manuscript must describe a technically sound piece of scientific research with data that supports the conclusions. Experiments must have been conducted rigorously, with appropriate controls, replication, and sample sizes. The conclusions must be drawn appropriately based on the data presented.

Reviewer #1: Partly

Reviewer #2: Yes

2. Has the statistical analysis been performed appropriately and rigorously?

Reviewer #1: Yes

Reviewer #2: Yes

3. Have the authors made all data underlying the findings in their manuscript fully available?

The [PLOS Data policy](https://urldefense.com/v3/__http:/track.editorialmanager.com/CL0/http:*2F*2Fwww.plosone.org*2Fstatic*2Fpolicies.action*23sharing/1/010f01990e0e91cd-74e4c46e-b743-4c4d-8143-59488c57b46a-000000/AfvEndC08xKY1ZfZNEV3YQZvLFM7RCrPIMiX5xSBO88=227__;JSUlJSU!!LQC6Cpwp!sqGHAxTi2C6t2-Xb-rykykSRTK2aFxyM8Bt5VMUuzCyaFHieJrJkZfuF76nCfujKuZdR1l-mcKVE1XGB_V0UeXIXZA$) requires authors to make all data underlying the findings described in their manuscript fully available without restriction, with rare exception (please refer to the Data Availability Statement in the manuscript PDF file). The data should be provided as part of the manuscript or its supporting information, or deposited to a public repository. For example, in addition to summary statistics, the data points behind means, medians and variance measures should be available. If there are restrictions on publicly sharing data—e.g. participant privacy or use of data from a third party—those must be specified.

Reviewer #1: Yes

Reviewer #2: Yes

4. Is the manuscript presented in an intelligible fashion and written in standard English?

PLOS ONE does not copyedit accepted manuscripts, so the language in submitted articles must be clear, correct, and unambiguous. Any typographical or grammatical errors should be corrected at revision, so please note any specific errors here.

Reviewer #1: Yes

Reviewer #2: Yes

5. Review Comments to the Author

Please use the space provided to explain your answers to the questions above. You may also include additional comments for the author, including concerns about dual publication, research ethics, or publication ethics. (Please upload your review as an attachment if it exceeds 20,000 characters)

**Reviewer #1:** Hello.
This is a good article and the topic chosen is important and special and has been studied in a proper way in this article.
I thank the authors.
I hope we will read more and better quality studies from them in the future.
Good luck.

**Author’s Response:** We would like to thank Reviewer #1 for their comments regarding the value of our study.

**Reviewer #2:** The study titled ‘Case-control study of autonomic symptoms in the setting of long COVID with tilt table testing’ by Durstenfeld et al. presents several intriguing findings. To further strengthen the manuscript, I recommend the following revisions:

**Authors’ Response:** Thank you for your interest in our manuscript and these helpful suggestions.

1. Page 3, Introduction Section: The introduction outlines prior findings and notes inconsistencies in the literature; however, it does not clearly highlight why this represents a significant clinical or scientific problem worth investigating. Beyond mentioning variability in prevalence estimates, the rationale for the study is not compelling. The authors should better articulate the importance of clarifying autonomic dysfunction in Long COVID, to more convincingly justify the need for the present study.

**Authors’ Response:** Thank you for asking us to highlight the significance of this problem and the justification for our study. We have rewritten the introduction to focus on clinical significance of the possible connection between autonomic symptoms and Long COVID. The new first paragraph of the introduction is as follows (Lines 45-55):

Autonomic disorders including postural orthostatic tachycardia syndrome (POTS), orthostatic hypotension (OH), and orthostatic intolerance without tachycardia or hypotension have been reported following COVID-19 as post-acute sequelae of COVID-19 or symptomatic Long COVID [1, 2]. The role of autonomic dysfunction as a contributor to Long COVID symptomatology is uncertain, but there is overlap between common Long COVID symptoms and common autonomic symptoms. A survey of individuals recruited through Long COVID support groups reported that two thirds of survey participants had a Composite Autonomic Symptom 31 (COMPASS-31) score >20, suggestive of autonomic dysfunction [3]. More severe autonomic symptoms are associated with worse quality of life and higher levels of disability among those with Long COVID [4]. Orthostatic intolerance is included in the diagnostic criteria for myalgic encephalitis/chronic fatigue syndrome (ME/CFS), which overlaps with Long COVID especially in its most disabling forms [5].

2. Page 3, Lines 51-55: When discussing the prior case series, the manuscript briefly notes the influence of beta blocker use and nitroglycerin provocation on HUTT outcomes. However, this limitation is not sufficiently emphasized. The authors should explicitly state that these methodological issues undermine the validity of the reported prevalence of orthostatic intolerance and contribute to inconsistencies across previous studies. This clarification is important to strengthen the rationale for why the present case-control study is warranted.

**Authors’ Response:** We have expanded the introduction to highlight the methodologic concerns and limitations of the prior studies (new Lines 56-70, with new text bold and underlined):

Head up tilt-table testing (HUTT) is the gold-standard **objective** test for assessing orthostatic symptoms in the setting of autonomic dysfunction, **but existing studies have major limitations**. A case-series of 24 individuals with cardiovascular phenotype post-acute sequelae of COVID-19, or Long COVID, referred from a Long COVID clinic, reported that 23/24 had “orthostatic intolerance” during HUTT. In that study, 15 (65%) had normal tests prior to administration of nitroglycerin and 22% were on beta blockers [6]. Beta blockers lower sensitivity and nitroglycerin lowers specificity for clinical symptoms [7]. Importantly, all 15 with nitroglycerin-provoked orthostatic intolerance reported that their Long COVID symptoms were improving or resolved, suggesting that pharmacologic provocation may not be the most clinically useful approach. **Both beta blockers and nitroglycerin modify physiologic responses to orthostatic stressors, so the relevance of this study’s findings on the mechanisms of symptoms in Long COVID is challenging to interpret.**

3. Page 3, Lines 58-60: When comparing prior studies, the statement that “other studies in different populations and using different methodologies have reported 0–30% meeting criteria for POTS” is too general. For a meaningful comparison, the authors should at least specify what these populations were and briefly note the study contexts, so that readers can better understand the reasons for variability and how the present study fits within the existing literature.

**Author’s Response:** We have expanded the introduction to highlight the methodologic concerns and limitations of the prior studies (new Lines 71-76, with new text bold and underlined):

Other studies in different populations and using different methodologies have reported that 0-30% of individuals met criteria for POTS [8-11]. **Those studies which reported higher prevalences were conducted in referral centers for autonomic dysfunction among individuals referred for clinical concern for Long COVID and/or specifically for autonomic dysfunction [10, 11]. Several studies used the active stand test or shortened 10-minute head up tilt testing [8, 9, 11].** In the context of the considerable variability of the findings reported, we conducted a case-control study of individuals with Long COVID, with and without postural symptoms consistent with autonomic dysfunction, compared to individuals who had recovered from COVID.

4. Pages 5-6, Lines 101-130: The HUTT protocol does not state whether participants were fasting. Please clarify, as recent food or caffeine intake could affect autonomic responses.
5. Pages 5-6, Head up tilt table testing: The HUTT includes several observer-dependent measurements during HUTT. It is unclear whether these were performed by a single investigator or multiple individuals. The authors should clarify this.

**Authors’ response:** We now clarify in Lines 118-119: “We conducted unmedicated head up tilt table testing (HUTT). Participants were instructed to fast and abstain from caffeine and nicotine.” Later we describe in Lines 134-135, “HUTT was performed and symptoms were recorded by trained nursing staff under supervision of cardiac electrophysiologists.” We also clarify that all interpretations were ultimately adjudicated by the first author in Line 149-150: “All studies were reviewed immediately by the supervising electrophysiologists and then subsequently by the first author (MSD) to apply the research case definitions.”

6. Table 1: Some variables are reported as mean, while others are reported as median. This distinction is not noted in the footnotes. For clarity, the footnotes should specify which variables are summarized by mean ± SD and which by median (IQR).

**Authors’ Response:** We have added footnotes A, B, C to specify this. We report the mean and standard deviation for normally distributed variables (ie age, HR, BP), and the median and interquartile range for skewed variables (ie COMPASS 31 score).

7. Page 13, Lines 255-266: While the authors highlight some methodological concerns in prior studies reporting POTS post-COVID, the discussion would benefit from a more detailed explanation of these issues. For example, the limitations of the active stand test and the implications of returning participants to supine before a full heart rate response occurs should be elaborated to help readers better understand why those studies may have underestimated or misclassified POTS.

**Authors’ Response:** We have substantially revised the discussion to expand on these issues. We also now mention three studies that used the NASA Lean test and now discuss some of the controversy over the best way to identify POTS including differences between active stand (or NASA lean) and HUTT.

6. PLOS authors have the option to publish the peer review history of their article ([what does this mean?](https://urldefense.com/v3/__https:/track.editorialmanager.com/CL0/https:*2F*2Fjournals.plos.org*2Fplosone*2Fs*2Feditorial-and-peer-review-process*23loc-peer-review-history/1/010f01990e0e91cd-74e4c46e-b743-4c4d-8143-59488c57b46a-000000/wYgZKAMNoYBHcAy3E3mex2zgw1CsqFuIGbbHnfJzU3Y=227__;JSUlJSUl!!LQC6Cpwp!sqGHAxTi2C6t2-Xb-rykykSRTK2aFxyM8Bt5VMUuzCyaFHieJrJkZfuF76nCfujKuZdR1l-mcKVE1XGB_V2iq2S6Hw$)). If published, this will include your full peer review and any attached files.


If you choose “no”, your identity will remain anonymous but your review may still be made public.


**Do you want your identity to be public for this peer review?** For information about this choice, including consent withdrawal, please see our [Privacy Policy](https://urldefense.com/v3/__https:/track.editorialmanager.com/CL0/https:*2F*2Fwww.plos.org*2Fprivacy-policy/1/010f01990e0e91cd-74e4c46e-b743-4c4d-8143-59488c57b46a-000000/PMqA0SrLplvGS5tt_af8q_c190QCwGVwXboznPRrrS0=227__;JSUl!!LQC6Cpwp!sqGHAxTi2C6t2-Xb-rykykSRTK2aFxyM8Bt5VMUuzCyaFHieJrJkZfuF76nCfujKuZdR1l-mcKVE1XGB_V1vo5_G4g$).

Reviewer #1: No

Reviewer #2: No
